# Laser-Induced Breakdown Spectroscopy (LIBS) for Monitoring the Formation of Hydroxyapatite Porous Layers

**DOI:** 10.3390/ma10121395

**Published:** 2017-12-06

**Authors:** Daniel Sola, Daniel Paulés, Lorena Grima, Jesús Anzano

**Affiliations:** 1 Laboratorio de Óptica, Centro de Investigación en Óptica y Nanofísica, Universidad de Murcia, Campus Espinardo, 30.100 Murcia, Spain; 2 Laboratorio Láser, Dpto. Química Analítica, Universidad de Zaragoza, 50.009 Zaragoza, Spain; danipaules25@gmail.com (D.P.); janzano@unizar.es (J.A.); 3 Instituto de Ciencia de Materiales de Aragón, Dpto. Ciencia y Tecnología de Materiales y Fluidos, Universidad de Zaragoza-CSIC, 50.018 Zaragoza, Spain; lgrima@unizar.es

**Keywords:** laser-induced breakdown spectroscopy, eutectic glass, bioactive materials

## Abstract

Laser-induced breakdown spectroscopy (LIBS) is applied to characterize the formation of porous hydroxyapatite layers on the surface of 0.8CaSiO_3_-0.2Ca_3_(PO_4_)_2_ biocompatible eutectic glass immersed in simulated body fluid (SBF). Compositional and structural characterization analyses were also conducted by field emission scanning electron microscopy (FESEM), energy dispersive X-ray spectroscopy (EDX), and micro-Raman spectroscopy.

## 1. Introduction

A new era for tissue engineering has emerged since the discovery of a bioactive glass by Hench et al. in 1970 [1]. In particular, silicon and silicon calcium phosphate materials have attracted scientist’s attention for being used as scaffolds in orthopaedic, oral, and maxillofacial applications. These materials, during exposure to simulated body fluid (SBF), develop a hydroxyapatite (HA) layer on their surface [2]. This reaction starts on the surface and usually leads to harmful shear-stress [3]. To enhance the ingrowth and the bioactivity of the ceramic implant, a suitable interconnected porous structure network is commonly utilized, which also provides a higher bioactivity rate and improves both the anchoring of the prosthesis and the blood and nutrition supply for the ingrowth of the new bone [4,5,6,7]. 

A novel path to accomplish in situ interconnected pore networks has been developed departing from dense bioactive and resorbable eutectic glass-ceramic and glasses of the CaO-SiO_2_-P_2_O_5_ and CaO-SiO_2_-P_2_O_5_-MgO systems [8,9,10]. Directionally-solidified eutectic ceramic (DSEC) oxides are composite ceramics obtained departing from a melt. Solidification conditions allow controlling the resulting microstructure and, hence, the material properties, making them suitable for structural and functional applications [11,12,13,14,15,16]. In particular, the CaSiO_3_/Ca_3_(PO_4_)_2_ eutectic composite shows remarkable optical properties. Its spectroscopic properties can be used as optical probes for biomedical applications [17,18,19]. Additionally, optical waveguides inscribed in this eutectic glass by means of the ultrafast laser inscription technique suggested a great potential for being used as integrated photonic devices, optical amplifiers, and laser sources [20,21]. Furthermore, this eutectic binary composite presents bioactive characteristics since wollastonite (W), CaSiO_3_, is bioactive with osteostimulative properties and tricalcium phosphate (TCP), Ca_3_(PO_4_)_2_, is osteoconductive and bioactive (resorbable) [22,23]. Both in vitro and in vivo biocompatibility, and bioactivity of this eutectic composite, have been widely studied for the last two decades [8,9,10,22,23,24,25,26,27]. 

In this work, we report on the characterization of the hydroxyapatite porous layer developed on the surface of the W-TCP eutectic glass after being immersed in SBF by using the laser-induced breakdown spectroscopy (LIBS) technique that is based on the generation of micro-plasma and emission spectroscopy measurements. In this technique, a high-energetic laser pulse, used as an atomization and excitation source, is directly focused on the sample surface and the formed plasma is analysed to obtain the multi-elemental composition of samples. LIBS is a single step, fast, robust, and stable technique with high spatial resolution, which can be carried out under atmospheric conditions [28]. In addition, sample preparation is not required, thus, providing a wide range of advantages when compared to other analytical techniques [29,30,31,32,33]. It is well-known that the W-TCP eutectic composite is capable of rearranging its morphology when it is soaked in human parotid saliva (HPS) or SBF so that the W phase is dissolved and the TCP phase undergoes a pseudomorphic transformation into HA [8,9,23,24]. Hence, the dense W-TCP ceramic is turned into a HA porous layer. The principal aim of this work is to assess the Si content of the sample surface by LIBS analysis to confirm the absence of this element in the layer generated after the sample being soaked into SBF and to conduct compositional and structural characterization analyses by field emission scanning electron microscopy (FESEM), energy dispersive X-ray spectroscopy (EDX) and micro-Raman spectroscopy to corroborate the presence of HA.

## 2. Experimental

### 2.1. Sample Fabrication

Eutectic glass samples were manufactured by means of the laser floating zone (LFZ) technique. This technique has been described in detail elsewhere [11,34,35]. For this purpose, tricalcium phosphate and wollastonite powders were mixed in the eutectic 20% Ca_3_(PO_4_)_2_, 80% CaSiO_3_ mol % composition. The resulting powders were isostatically pressed at 200 MPa for 2 min to obtain ceramic rods which were sintered at 1200 °C for 10 h. Samples were grown in air and annealed at 650 °C for 5 h to relieve inner stresses. The development of the HA layer on the surface of the glass samples was carried out by soaking, for a one-month period, a glass sample in SBF, prepared according to the standard process [36]. The sample was kept at the human body temperature of 37 °C by means of a Memmer Beschickung-loading-model 100–800 stove (Memmert GmbH, Schwabach, Germany).

### 2.2. Characterization Techniques

LIBS characterization was carried out by means of a Q-switched Nd:YAG laser (Brilliant Quantel, model Ultra CFR, Les Ulis Cedex, France) with emission at 1064 nm, emitting 7.7 ns laser pulses with 50 mJ maximum pulse energy. Plasma emission was collected by using a bifurcated optical fiber (QBIF600-UV-VIS, 600 μm, Premium Bifurcated Fiber, UV-VIS, 2 m, ATO, Largo, FL, USA) adjusted at 45° to the sample surface and connected to a dual-channel Ocean-Optics spectrometer (LIBS 2500plus, Ocean Optics Inc., Dunedin, FL, USA). The laser beam was directly focused on the surface of the samples through a 150 mm focal length lens. In order to avoid detector saturation, pulse energy and irradiance were set at 30 mJ and 73.5 MW/cm^2^, respectively.

Semi-quantitative compositional analysis and morphology were characterized by means of field emission scanning electron microscopy (FESEM) using a Carl Zeiss MERLIN microscope with an incorporated energy dispersive X-ray detector (EDX) (Carl Zeiss microscopy GmbH, Munich, Germany). X-ray diffraction (XRD) analyses were carried out to determine the amorphous character of glass samples by means of a Bruker D8 Advance diffractometer (Bruker, Billerica, MA, USA). Raman dispersion measurements were performed using a confocal Raman spectrometer (Witec Alpha 300 M+) (Witec, Ulm, Germany) equipped with a thermoelectric-cooled CCD detector. As the excitation source, a 488 nm laser was used and the scattered light was collected through a 50× microscope objective lens. The output power of the laser was kept below 1 mW in order to avoid significant local heating of the sample. 

## 3. Results and Discussion

**Figure 1:**
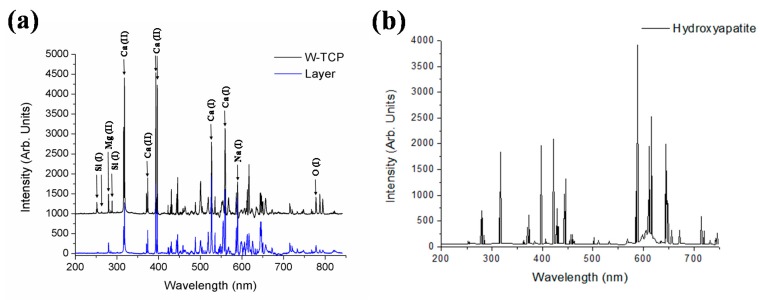
Comparative LIBS spectra between the W-TCP eutectic glass and the layer produced on the surface of the sample (**a**). The spectra shown are the average of 10 shots with an integration time of 3 ms. The HA LIBS spectrum is also presented for comparison purposes (**b**).

Figure 1 shows LIBS spectra recorded in the spectral range of 200–850 nm for both the W-TCP eutectic glass and layer developed on the sample surface after being soaked into SBF for one month. The LIBS spectrum of HA is also presented for comparison purposes [37]. The spectra show strong characteristic emission lines that can be assigned according to the National Institute of Standards and Technology (NIST). The main atomic emission lines corresponding to Si (I), Ca (I), Ca (II), Mg (II), Na (I), and O (I) are pointed out in the figure and the assigned wavelengths are listed in Table 1. When the dense W-TCP eutectic glass is immersed in SBF, the reaction of the material with the SBF gives rise to a porous layer of HA, which finally covers the surface of the sample. It is well known that for a glass to be bioactive and, hence, to bond to bone, a calcium phosphate layer must form at its surface. The mechanisms of this reaction were proposed by Hench et al. [1,2], and can be summarized in the following five stages: (i) rapid exchange of alkali or alkali-earth ions with H^+^ or H_3_O^+^ from solution; (ii) loss of soluble silica in the form of Si(OH)_4_ to the solution; (iii) condensation and repolymerization of SiO_2_-rich layer on the surface depleted in alkalis and alkaline-earth cations; (iv) migration of Ca^2+^ and PO_4_^3−^ groups to the surface through the SiO_2_-rich layer forming a CaO–P_2_O_5_-rich film on top of the SiO_2_-rich layer, followed by the growth of the amorphous CaO–P_2_O_5_-rich film by incorporation of soluble calcium and phosphorous from solution; and (v) crystallization of the amorphous CaO–P_2_O_5_ film by incorporation of OH^−^ anions from solution to form a hydroxyapatite layer.

**Figure 2:**
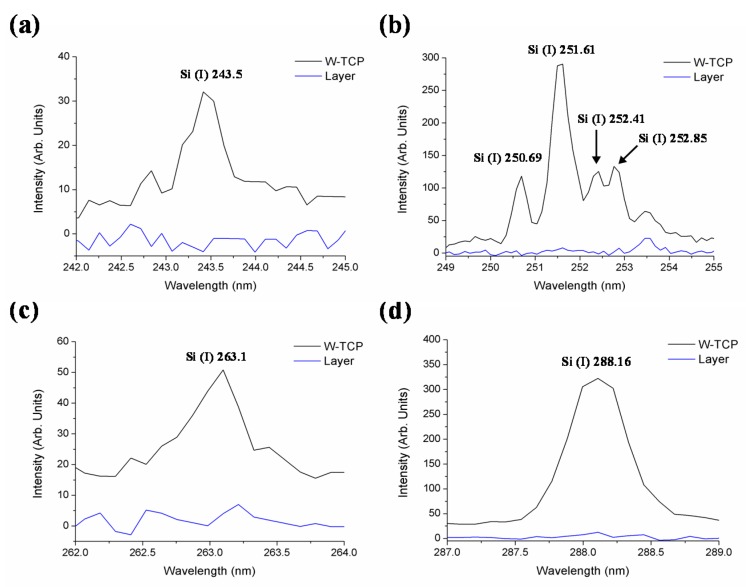
LIBS spectral signals of Si (I) in both the W-TCP eutectic glass and the HA layer at 243.5 nm (**a**), 250.69, 251.61, 252.41, 252.85 nm (**b**), 263.1 (**c**) and 288.16 (**d**).

Figure 2 shows LIBS spectral emission lines of Si (I) at 243.5 (a), 250.69, 251.61, 252.41, 252.85 (b), 263.1 (c), and 288.16 (d). It can be observed that none of these spectral emission signals appeared in the layer produced after immersion in SBF, thus confirming the absence of silicon in the layer developed on the surface of the sample. 

**Figure 3:**
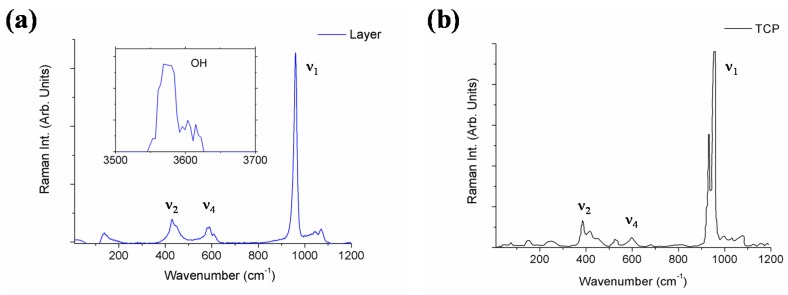
Micro-Raman spectra collected on the surface of the sample after a one-month immersion in SBF (**a**). The standard TCP Raman spectrum is also presented for comparison purposes (**b**).

Next, both the chemical composition and structure of the layer produced on the surface of the sample after a one-month immersion in SBF was investigated by micro-Raman spectroscopy for two wavenumber regions; 50–1200 cm^−1^ and 3500–3700 cm^−1^ (Figure 3). The Raman spectrum of standard TCP is also presented for comparison purposes. The Raman spectra collected were made up of sharp peaks and broad bands which can be assigned to the HA Raman spectra previously reported in the scientific literature [9,26]; a narrow intense peak located at 962 cm^−1^, corresponding to symmetric stretching of PO_4_^3−^ modes, and broad bands at 400–500, 570–625 and 1020–1095 cm^−1^ are attributed, respectively, to ν_2_^−^, ν_4_^−^, and ν_3_^−^ type internal PO_4_^3−^ modes, and a strong sharp peak located at 3576 cm^−1^ is assigned to the O–H stretching mode. It is worth highlighting that HA Raman spectra show significant variations when compared to TCP spectra, the most relevant of which is that the O–H stretching mode did not appear. Therefore, micro-Raman analyses carried out on the layer developed on the sample surface confirmed the generation of a HA layer. 

**Figure 4:**
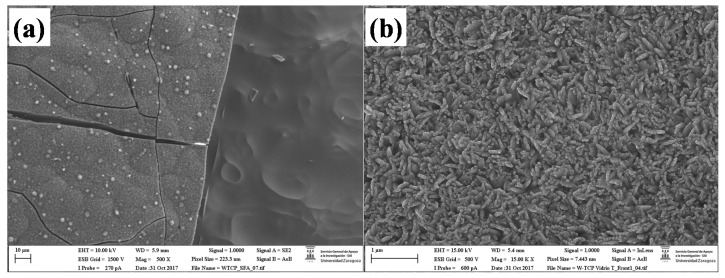
SEM micrographs of the W-TCP eutectic sample after a one-month immersion in SBF. General view (**a**), and a detail of the layer developed on the surface (**b**).

Finally, microstructural and semi-quantitative chemical composition analyses were carried out by SEM-EDX aiming at analysing the morphology of the sample, determining the elements both the glass and the layer were comprised of, and the Ca/P ratio of the layer. Figure 4 shows a general view micrograph of the sample soaked in SBF (a) and a detail of the layer (b). SEM observation showed that a new layer was formed on the surface of the samples, which consisted of HA nanocrystals, fibrillar in shape, and randomly oriented, thus providing porosity to the new surface. The cracks observed revealed that the coating formed had different properties than the parent glass, as cracks were not present on starting samples. The EDX spectrum shown in Table 2 indicates that the composition of the glass is close to the theoretical value. In addition, these analyses revealed the formation on the surface of the glass of a layer rich in Ca and P, with a Ca/P ratio of about 1.3. It is worth mentioning that these analyses corroborated that Si was not present in the layer. Thus, during immersion, the bioactive glass surface dissolved and a new surface formed by precipitation and transformation reactions leading to a crystallized, Ca-deficient apatite, similar to bone in its composition.

## 4. Conclusions

W-TCP eutectic glasses were soaked in simulated body fluid for a one-month period of time in which a hydroxyapatite porous layer was developed on the surface. Laser-induced breakdown spectroscopy (LIBS) spectra acquired on the sample surface showed that Si (I) emission lines were not present in the layer developed after the immersion period. Micro-Raman spectroscopy analyses carried out on the surface confirmed the crystalline nature of this layer, the Raman spectra of which corresponded to hydroxyapatite. Finally, SEM-EDX characterization indicated that the layer composition was rich in Ca and P with a Ca/P ratio around 1.3 and at the same time corroborating the Si absence on the layer. 

## Figures and Tables

**Table 1:** List of the main spectral lines observed in the samples.

Element	Sample	Wavelength (nm)
Si (I)	W-TCP	243.5, 250.69, 251.61, 252.41, 252.85, 263.1, 288.16
Ca (I)	W-TCP, HA	527.03, 559.45
Ca (II)	W-TCP, HA	317.93, 373.69, 393.37, 396.85
Mg (II)	W-TCP, HA	279.55
Na (I)	W-TCP, HA	589
O (I)	W-TCP, HA	777.5

**Table 2:** EDX compositional analysis in at % of both the W-TCP glass and surface layer.

Sample	Si	P	Ca
Glass	12.73	6.27	18.86
Layer	–	16.56	21.70

## References

[1] Hench L.L., Splinter R.J., Greenle T.K., Allen W.C. (1971). Bonding mechanisms at the interface of ceramic prosthetic materials. J. Biomed. Mater. Res..

[2] Hench L.L., Wilson J. (2013). An Introduction to Bioceramics.

[3] Best S.M., Porter A.E., Thian E.S., Huan J. (2008). Bioceramics: Past, present and for the future. J. Eur. Ceram. Soc..

[4] Lu J.X., Flautre B., Anselme K., Hardouin P., Gallur A., Descamps M., Thierry B. (1999). Role of interconnections in porous bioceramics on bone recolonization in vitro and in vivo. J. Mater. Sci. Mater. Med..

[5] De Groot K., Le Geros R., Ducheyne P. (1988). Significance of Porosity and Physical Chemistry of Calcium Phosphate Ceramics.

[6] Von Doernberg M.C., von Rechenberg B., Bohner M., Grunenfelder S., van Lenthe G.H., Muller R., Gasser B., Mathys R., Baroud G., Auer J. (2006). In vivo behaviour of calcium phosphate scaffolds with four different pore sizes. Biomaterials.

[7] Bungo O., Mitsuru T., Shunsuke F., Masashi N., Tadashi K., Takashi N. (2006). Pore throat size and connectivity determine bone and tissue ingrowth into porous implants: Tree-dimensional micro-CT based structural analyses of porous bioactive titanium implants. Biomaterial.

[8] De Aza P.N., Guitian F., de Aza S. (1995). Phase diagram of wollastonite-tricalcium phosphate. J. Am. Ceram. Soc..

[9] De Aza P.N., Guitian F., de Aza S. (1997). Bioeutectic: A new ceramic material for human bone replacement. Biomaterials.

[10] Magallanes-Perdomo M., de Aza A.H., Sobrados I., Sanz J., Pena P. (2012). Structure and properties of bioactive eutectic glasses based on the Ca_3_(PO_4_)_2_–CaSiO_3_–CaMg(SiO_3_)_2_ system. Acta Biomater..

[11] Llorca J., Orera V.M. (2006). Directionally-solidified eutectic ceramic oxides. Prog. Mater. Sci..

[12] Sola D., Ester F.J., Oliete P.B., Peña J.I. (2011). Study of the stability of the molten zone and the stresses induced during the growth of Al_2_O_3_–Y_3_Al_5_O_12_ eutectic composite by the laser floating zone technique. J. Eur. Ceram. Soc..

[13] Ester F.J., Sola D., Peña J.I. (2008). Thermal stresses in the Al_2_O_3_-ZrO_2_(Y_2_O_3_) eutectic composite during the growth by the laser floating zone technique. Bol. Soc. Esp. Ceram..

[14] Sola D., Peña J.I. (2012). Laser machining of Al_2_O_3_-ZrO_2_ (3%Y_2_O_3_) eutectic composite. J. Eur. Ceram. Soc..

[15] Orera V.M., Peña J.I., Merino R.I., Lazaro J.A., Valles J.A., Rebolledo M.A. (1997). Prospects of new planar optical waveguides based on the eutectic microcomposites of insulating crystals: ZrO_2_ (c)-CaZrO_3_ erbium doped system. Appl. Phys. Lett..

[16] Sola D., Oliete P.B., Peña J.I. (2016). Directionally solidified fabrication in planar geometry of Al_2_O_3_-Er_3_Al_5_O_12_ eutectic composite for thermophotovoltaic devices. Opt. Express.

[17] Pardo J.A., Peña J.I., Merino R.I., Cases R., Larrea A., Orera V.M. (2002). Spectroscopic properties of Er^3+^ and Nd^3+^ doped glasses with 0.8CaSiO_3_–0.2Ca_3_(PO_4_)_2_ eutectic composition. J. Non-Cryst. Solids.

[18] Sola D., Balda R., Peña J.I., Fernandez J. (2012). Site-selective laser spectroscopy of Nd^3+^ ions in 0.8CaSiO_3_–0.2Ca_3_(PO_4_)_2_ biocompatible eutectic glass-ceramics. Opt. Express.

[19] Sola D., Balda R., Al-Saleh M., Peña J.I., Fernandez J. (2013). Time-resolved fluorescence line-narrowing of Eu^3+^ in biocompatible eutectic glass-ceramics. Opt. Express.

[20] De Mendivil J.M., Sola D., de Aldana J.R.V., Lifante G., De Aza A.H., Pena P., Peña J.I. (2015). Ultrafast direct laser writing of cladding waveguides in the 0.8CaSiO_3_–0.2Ca_3_(PO_4_)_2_ eutectic glass doped with Nd^3+^ ions. J. Appl. Phys..

[21] Sola D., De Mendibil J.M., De Aldana J.R.V., Lifante G., Balda R., De Aza A.H., Pena P., Fernandez J. (2013). Stress-induced buried waveguides in the 0.8CaSiO_3_–0.2Ca _3_(PO_4_)_2_ eutectic glass doped with Nd^3+^ ions. Appl. Surf. Sci..

[22] Carrodeguas R.G., de Aza S. (2011). α-Tricalcium phosphate: Synthesis, properties and biomedical applications. Acta Biomater..

[23] De Aza P.N., Luklinska Z.B., Anseau M.R., Hector M., Guitian F., De Aza S. (2000). Reactivity of a wollastonite-tricalcium phosphate Bioeutectic^®^ ceramic in human parotid saliva. Biomaterials.

[24] De Aza P.N., Guitian F., de Aza S. (1998). A new bioactive material which transforms in situ into hydroxyapatite. Acta Mater..

[25] Magallanes-Perdomo M., Pena P., de Aza P.N., Carrodeguas R.G., Rodríguez M.A., Turrillas X., de Aza S., de Aza A.H. (2009). Devitrification studies of wollastonite–tricalcium phosphate eutectic glass. Acta Biomater..

[26] De Aza P.N., Peña J.I., Luklinska Z.B., Meseguer-Olmo L. (2014). Bioeutectic^®^ ceramics for biomedical application obtained by Laser Floating Zone method. In vivo Evaluation. Materials.

[27] Magallanes-Perdomo M., De Aza A.H., Sobrados I., Sanz J., Luklinska Z.B., Pena P. (2017). Structural changes during crystallization of apatite and wollastonite in the eutectic glass of Ca_3_(PO_4_)_2_-CaSiO_3_ system. J. Am. Ceram. Soc..

[28] Paules D., Hamida S., Lasheras R.J., Escudero M., Benouali D., Cáceres J.O., Anzano J. (2018). Characterization of natural and treated diatomite by Laser-Induced Breakdown Spectroscopy (LIBS). Microchem. J..

[29] Cremers D.A., Radziemski L.J. (2013). Handbook of Laser-Induced Breakdown Spectroscopy.

[30] Hahn D.W., Omenetto N. (2010). Laser-Induced Breakdown Spectroscopy (LIBS), Part I: Review of Basic Diagnostics and Plasma-Particle Interactions: Still-Challenging Issues within the Analytical Plasma Community. Appl. Spectrosc..

[31] Hahn D.W., Omenetto N. (2012). Laser-Induced Breakdown Spectroscopy (LIBS), Part II: Review of Instrumental and Methodological Approaches to Material Analysis and Applications to Different Fields. Appl. Spectrosc..

[32] Miziolek A.W., Palleschi V., Schechter I. (2006). Laser-Induced Breakdown Spectroscopy (LIBS). Fundamentals and Applications.

[33] Parigger C.G. (2013). Atomic and molecular emissions in laser-induced breakdown spectroscopy. Spectrochim. Acta Part B At. Spectrosc..

[34] Sola D., Conejos D., de Mendivil J.M., Ortega-San-Martin L., Lifante G., Peña J.I. (2015). Directional solidification, thermo-mechanical and optical properties of (Mg_x_Ca_1−x_)_3_Al_2_Si_3_O_12_ glasses doped with Nd^3+^ ions. Opt. Express.

[35] Arias-Egido E., Sola D., Pardo J.A., Martínez J.I., Cases R., Peña J.I. (2016). On the control of optical transmission of aluminosilicate glasses manufactured by the laser floating zone technique. Opt. Mater. Express.

[36] Oyane A., Kim H., Furuya T., Kokubo T., Miyazaki T., Nakamura T. (2003). Preparation and assessment of revised simulated body fluid. J. Biomed. Mater. Res. A.

[37] Tariq U., Haider Z., Hussain R., Tufail K., Ali J. LIBS Analysis of Hydroxyapatite Extracted from Bovine Bone for Ca/P Ratio Measurements. Proceedings of the 9th International Conference on Plasma Science and Applications.

